# Increased Serum Levels and Chondrocyte Expression of Nesfatin-1 in Patients with Osteoarthritis and Its Relation with BMI, hsCRP, and IL-18

**DOI:** 10.1155/2013/631251

**Published:** 2013-10-23

**Authors:** Lifeng Jiang, Jiapeng Bao, Xindie Zhou, Yan Xiong, Lidong Wu

**Affiliations:** Department of Orthopedics Surgery, The Second Hospital of Medical College, Zhejiang University, Hangzhou 310000, China

## Abstract

*Background*. Adipokines have been proved to relate with osteoarthritis (OA). As a recently discovered adipokine, nesfatin-1 relationship with OA has not been reported. *Aim*. To determine the levels of nesfatin-1 in serum and synovial fluid (SF) from patients with and without OA; to examine the correlation between nesfatin-1 levels and high sensitivity C-reactive protein (hsCRP), Type IIA Collagen N Propeptide (PIIANP), and IL-18 (interleukin-18) levels in serum or synovial fluid. *Methods*. Serum and SF were collected from knee OA patients and healthy persons, respectively. Five articular tissues were obtained during TKR for immunohistochemistry (IHC). Nesfatin-1 levels, hsCRP, PIIANP, and IL-18 in serum and SF were analyzed by enzyme-linked immunosorbent assay (ELISA). *Results*. Nesfatin-1 gene was expressed in OA-affected articular cartilage. OA serum contained significantly higher levels of nesfatin-1, as compared to serum from healthy controls (*P* < 0.05), and nesfatin-1 levels in OA serum exceeded those in paired SF samples (*P* < 0.001). Significant correlation was found between serum nesfatin-1 and hsCRP levels in OA patients (*r* = 0.593, *P* = 0.00005) and also synovial nesfatin-1 and IL-18 levels (*r* = 0.560, *P* = 0.0017). *Conclusion*. Nesfatin-1 is present in articular tissues and may contribute to the physiopathologic changes in OA. Nesfatin-1, accompanied with hsCRP and IL-18, could be new molecular makers to speculate OA progression.

## 1. Introduction 

Osteoarthritis (OA) is a slowly progressive degenerative disease characterized by escalated loss of articular cartilage. It is one of the most common causes of pain and disability in middle-aged and older people. The major pathological changes during the progression of OA are articular cartilage breakdown, osteophyte formation, subchondral sclerosis, and other alterations [1]. Local inflammation and synovitis are present in many patients with OA and have also been observed in animal models of OA [2]. In addition to mechanical factors such as obesity induced weight bearing, a lot of research has concentrated on biochemical and genetic factors that contribute to alterations of chondrocytes and synoviocytes.

Since the secretory functions of adipose tissue have been uncovered, adipokines have been linked to many diseases, such as cardiovascular disease, metabolic complications, atherosclerosis, and inflammatory and immune-related disorders [3–5]. Much recent literature suggests that adipokines play an important role in OA. Evidence suggests that adipokines including leptin, adiponectin, resistin and visfatin exert proinflammatory and catabolic/anabolic roles during the pathophysiology of OA [6–8]. No study has demonstrated the precise relation of the newly discovered adipokine nesfatin-1 with articular cartilage metabolism.

It has been reported that high sensitivity C-reactive protein (hsCRP) associates with local inflammatory in patients with OA and may reflect the progression [9, 10]. But some study demonstrated there was no significant relationship between CRP and incidence of knee or hip OA [11]. In patients with coronary artery disease, serum adiponectin concentration was negatively correlated with the hsCRP concentration [12]. Type IIA Collagen N Propeptide (PIIANP), a major collagen in cartilage, was found decreased in knee OA patients and associated with decrease severity [13, 14]. Interleukin-18 (IL-18) is a member of the IL-1 superfamily; as an inflammatory cytokine, IL-18 has the function of promoting cartilage loss [15]. As the typical adipokine, leptin could enhance the synthesis of proinflammatory mediators in human osteoarthritic cartilage [16]. For this background, we speculate whether nesfatin-1 has some relation with inflammatory mediators or collagen in cartilage.

Nesfatin-1 is an 82-amino-acid peptide that was first described in 2006 by Oh IS and colleagues from Maebashi, Japan [17]. They found a new anorexigenic protein derived from nucleobindin 2 (NUCB2) and named it NUCB2-encoded satiety and fat-influencing protein (nesfatin). NUCB2 had been reported to be a secreted protein of unknown function [18]. It produces three major peptide products: nesfatin-1 (spanning residues 1–82), nesfatin-2 (residues 85–163), and nesfatin-3 (residues 166–396). It is worth mentioning that nesfatin-1 is the only biologically active peptide known to be involved in food restriction [19]. So far, many researchers have focused on the neuroendocrine role of nesfatin-1. The appetite regulation of nesfatin-1 was first described: the intracerebroventricular injection of nesfatin-1 decreases food intake in a dose-dependent manner [20]. It was also reported that nesfatin-1 has a relation with mental disease. For example, Gunay et al. found decreased plasma nesfatin-1 levels in patients with anxiety [19]. In addition to the cardiovascular system, nesfatin-1 modulates blood pressure through effects on peripheral arterial resistance [21]. Zhang et al. discovered that nesfatin-1/NUCB2 IR cells are widespread in the rodent digestive organs such as the pancreas, stomach, and duodenum [22].

Recent evidence suggests that central nesfatin-expressing neurons are sensitive to peripheral inflammatory stimuli and could account for the reduction in food intake [23]. Furthermore, nesfatin-1 plays an anti-inflammatory and antiapoptotic role in subarachnoid hemorrhage-induced oxidative brain damage in rats [24]. Nesfatin-1 may inhibit the inflammatory response via NF-*κ*B signaling [25]. In light of the aforementioned studies, it is logical to hypothesize that nesfatin-1 could have a protective or harmful role in OA. The present study was performed to test this hypothesis.

The aim of this study was to investigate the expression profile of nesfatin-1 in synovial fluid (SF) and serum obtained from patients with OA and to compare with the serum levels of nesfatin-1 in healthy controls. In addition, we compared nesfatin-1 gene expression in articular cartilage in patients with OA and controls. We also measured the levels of hsCRP, PIIANP in serum and IL-18 in synovial, comparing the correlations with nesfatin-1 levels. Furthermore, with the aid of immunohistochemistry (IHC) we examined nesfatin-1 levels in five OA articular tissues (cartilage, osteophytes, synovium, meniscus, and the infrapatellar fat pad).

## 2. Materials and Methods

### 2.1. Patients and Samples

SF and serum were obtained from 40 patients with OA who underwent total knee replacement (TKR) (26 females and 14 males; mean age 66.17 ± 5.82 years; mean BMI 25.34 ± 2.81 kg/m^2^). Patients were diagnosed as having primary OA by both clinical and radiography. Based on BMI, patients were divided into three stratifications, BMI≦25 (22 patients), 25 < BMI≦30 (15 patients), BMI≧30 (3 patients). Normal serum samples were obtained from 25 healthy age-matched individuals without OA and other diseases (16 females and 9 males with mean age 61.52 ± 6.45 years, mean BMI 25.06 ± 1.71 kg/m^2^). All patients with clinical, intraoperative, or pathologic evidence of previous trauma, avascular necrosis, or inflammatory arthropathy were excluded. Patients with diabetes mellitus, coronary artery disease, and history of myocardial infarction, history of tobacco use within the last 5 years, history of recent trauma or recent infection were also excluded. Serum was obtained at the time of the patient admitted into hospital. Collected serum was centrifuged at 2000 ×g for 15 min and SF was centrifuged at 8000 ×g for 15 min. Serum and SF samples were then stored at −80°C. The personal and clinical characteristics of the patients and control individuals are summarized in Table 1.

OA-affected articular tissues were obtained from six patients who underwent TKR. Under aseptic conditions, samples were washed in ice-cold phosphate-buffered saline and five tissues (cartilage, osteophyte, synovium, meniscus, and infrapatellar fat pad) were separated and stored at −80°C for polymerase chain reaction (PCR) analysis. The rest of the samples were fixed in 4% buffered paraformaldehyde for IHC analysis. Other specimens of OA cartilage were collected from the femoral heads of eighteen patients with hip arthritis during total hip arthroplasty (THA) surgery. Normal femoral head cartilage was obtained from eighteen patients with femoral neck fractures without OA who underwent THA. All patients were treated at the Second Affiliated Hospital of Zhejiang University College of Medicine. This study was approved by the local ethics committee, and written informed consent was obtained from each volunteer.

### 2.2. Nesfatin-1 mRNA Measurement by Quantitative Real-Time PCR

#### 2.2.1. Nesfatin-1 Gene Expression in Knee Articular Tissues from Patients with OA

Five types of OA knee articular samples (cartilage, osteophyte, synovium, meniscus, infrapatellar fat pad) were pulverized in liquid nitrogen, and total RNA was extracted using TRIzol reagent (Sigma-Aldrich, St. Louis, MO, USA). First strand cDNA was synthesized from 1 *μ*g of total RNA using a Moloney murine leukemia virus reverse transcriptase cDNA synthesis kit (Promega, Madison, WI, USA). Quantitative real-time PCR was carried out using an iQTM SYBR Green premix ikPCR kit (Takara, Japan) and an iCycler system (Bio-Rad). The primers used are shown in Table 2. 18s RNA transcript (NR 003286) primers (forward, 5′-GACTCAACACGGGAAACCTCAC-3′; and reverse, 5′-CCAGACAAATCGCTCCACCAAC-3′) were used for parallel amplification of murine to normalize the expression data of the target transcripts. The relative expression levels of targeted genes were calculated for 100 copies of the 18s housekeeping gene using the formula: n=100×2-(ΔCT  targeted  gene - ΔCT 18s  RNA).

#### 2.2.2. Nesfatin-1 Gene Expression in Cartilage from Patients with Hip Arthritis and Femoral Neck Fractures

Samples were obtained from 18 patients with hip arthritis and eighteen age/BMI-matched patients with femoral neck fractures but without OA at the time of THA surgery. The samples were pulverized and RNA was extracted as described above. The target genes were the same as those mentioned above.

### 2.3. Nesfatin-1 Protein Measurement by ELISA

Nesfatin-1 concentrations in serum and SF were measured using a commercially available enzyme-linked immunoassay (ELISA) kit (Phoenix Peptides, Burlingame, CA, USA), according to the manufacturer's instructions. The ELISA kit is designed to measure the concentration of human nesfatin-1 (1–82) in human serum/plasma and conditioned medium. It can be used as long as the level of nesfatin-1 in the sample is above the sensitivity limit of the kit. Before assaying, serum samples were diluted with 1× assay buffer at room temperature (20–23°C). The dilution factor was 5 times. The enzyme-substrate reaction was terminated by the addition of stop solution.

### 2.4. Measurement of hsCRP, PIIANP, and IL-18

An hsCRP ELISA kit (Hemagen Diagnostics, Inc., Columbia, MD) was utilized with a lower limit of detection of 0.5 mg/L. Patient serum was diluted according to the manufacturer's directions. PIIANP ELISA kit (PIIANP, Linco, St. Louis, MO, USA) was used to measure serum PIIANP levels. This ELISA is based on a polyclonal antiserum raised against the recombinant human glutathione S transferase- (GST-)exon 2 fusion protein and uses recombinant human GST-exon 2 as a standard. The specificity of the antibody against PIIANP was previously demonstrated by Western blot analyses against the recombinant exon 2 protein (prior to and after cleavage with thrombin) and against type IIA procollagen isolated from the culture medium of human fetal ribs. IL-18 in synovial was also measured using an ELISA according to the manufacturer's instructions.

### 2.5. Immunohistochemical Analysis of Nesfatin-1 in Knee Cartilage, Osteophytes, and Synovium from Patients with OA

Postsurgical knee cartilage specimens obtained from three patients were fixed in 4% buffered paraformaldehyde for 2 days and decalcified with buffered 20% EDTA (pH 7.4). After dehydration and embedding in paraffin, 5 *μ*m thick sections were prepared, deparaffinized in xylene, and rehydrated in a graded ethanol series. Serial sections from each specimen were stained with hematoxylin and eosin and a rabbit antibody against human nesfatin-1. The following steps were performed automatically at 37°C using the Benchmark XT Slide Staining System (Ventana Medical Systems, Tucson, AZ, USA). Antigen retrieval was performed by immersing slides in citrate buffer (pH 6.0) for 15 minutes, and endogenous peroxidase activity was blocked through incubation with 1% H_2_O_2_ for 4 minutes. The sections were incubated with an anti-human nesfatin-1 receptor antibody at (dilution 1 : 100) for 60 minutes at room temperature. An UltraVision LP kit (Lab Vision, Fremont, CA, USA) was used to visualize the immunostaining. The slides were stained using a diaminobenzidine (DAB) detection kit and counterstained with hematoxylin.

### 2.6. Statistical Analyses

Assays were performed in triplicate. All data are expressed as the mean ± standard deviation (SD). Statistical analyses were performed with SPSS version 19.0 for Windows. BMI, gender, and age were adjusted. Statistically significant differences between matched SF and serum samples, OA serum and normal serum nesfatin-1 levels, nesfatin-1, hsCRP, and PIIANP levels in serum, nesfatin-1 and IL-18 levels in synovial, and nesfatin-1 gene expression in OA cartilage and cartilage from patients with femoral neck fractures were identified by paired Student's *t*-test and nonparametric test. Spearman's rank order correlation coefficient was used to examine the relationship between the above-mentioned index levels. Differences were considered significant when *P* was <0.05.

## 3. Results

### 3.1. Nesfatin-1 Expression in Five OA Knee Articular Tissues

**Figure 1:**
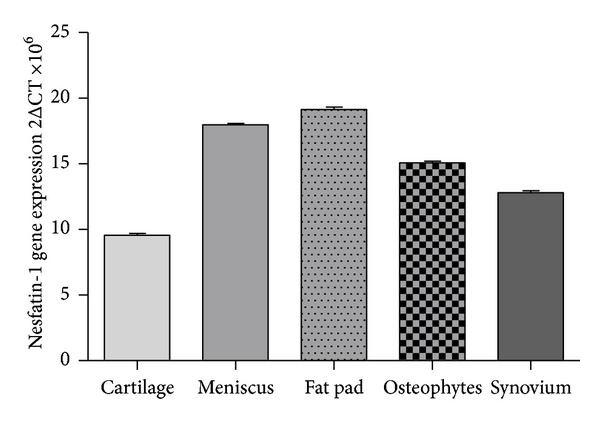
Nesfatin-1 gene expression in five OA-affected articular tissues. All the five OA affected cartilage, osteophytes, synovium, meniscus, and fat pad have nesfatin-1 gene expression.

To assess the gene expression of nesfatin-1 in OA-affected articular tissues, we examined cartilage, osteophytes, synovium, meniscus, and fat pad samples from six patients with OA who underwent TKR surgery by real-time quantitative PCR. Nesfatin-1 gene was expressed in all of five tissues (Figure 1).

### 3.2. Increased mRNA Expression of Nesfatin-1 in Cartilage from Patients with Hip Arthritis Compared with Cartilage from Patients with Femoral Neck Fractures

**Figure 2:**
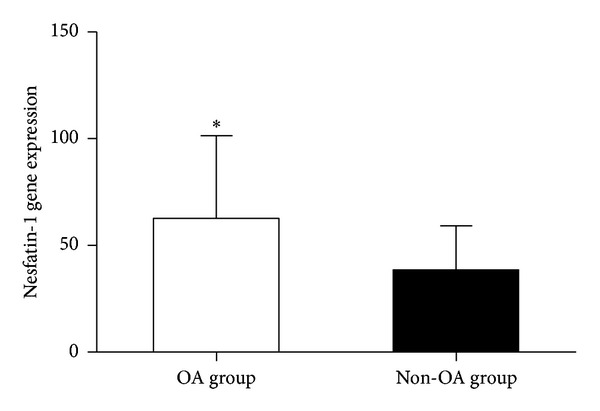
Nesfatin-1 gene expression in OA and non-OA cartilage. Nesfatin-1 mRNA levels were significantly higher in patients with OA than in femoral neck fracture controls (*P < 0.05).

We compared nesfatin-1 mRNA expression in OA cartilage and femoral neck fracture, non-OA cartilage by quantitative real-time PCR. Nesfatin-1 mRNA was detected in both types of cartilage; however, OA articular cartilage exhibited significantly higher nesfatin-1 expression compared with femoral neck fracture, non-OA cartilage (*P* < 0.05) (Figure 2).

### 3.3. Nesfatin-1 Levels in Serum and SF from Patients with OA and Healthy Controls

**Figure 3:**
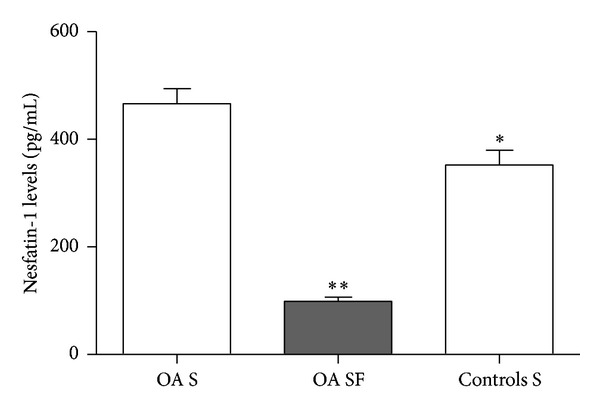
Nesfatin-1 levels in serum (S) and synovial fluid (SF). Serum nesfatin-1 levels were significantly higher in patients with OA than in controls (*P < 0.05). In patients with OA, nesfatin-1 levels were lower in SF than in paired serum samples (**P < 0.001).

OA serum contained significantly elevated levels of nesfatin-1 (466.45 ± 175.83 pg/mL, *n* = 40), as compared with serum from healthy controls (352.05 ± 137.51 pg/mL, *n* = 25) (*P* < 0.05). The mean nesfatin-1 level in SF from patients with OA was 98.96 ± 47.97 pg/mL. The nesfatin-1 level in serum was significantly higher than that in paired SF samples (*P* < 0.001) (Figure 3).

In the OA group, there were no sex differences in serum and SF nesfatin-1 levels. The serum nesfatin-1 concentration was 494.57 ± 191.83 pg/mL in females (*n* = 26) and 414.23 ± 132.13 pg/mL in males (*n* = 14) (*P* > 0.05), while the SF nesfatin-1 concentration was 94.59 ± 49.50 pg/mL in females and 107.05 ± 45.61 pg/mL in males (*P* > 0.05).

### 3.4. Different Nesfatin-1 Levels in Serum and Synovial of OA Patients with Different BMI

**Figure 4:**
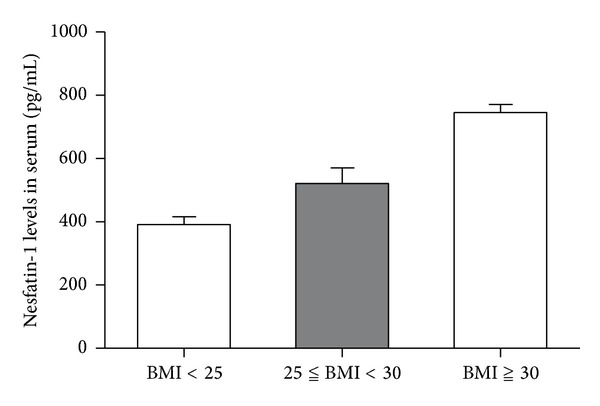
Different Nesfatin-1 levels in serum of OA patients with different BMI. On different BMI stratifications, the nesfatin-1 levels in serum, respectively, were 391.34 ± 115.81 (BMI≦25), 520.87 ± 192.41 (25 < BMI≦30), and 745.21 ± 45.24 (BMI≧30). Kruskal-Wallis test showed *χ* ^2^ = 13.59, *P* = 0.001.

On different BMI stratifications, the nesfatin-1 levels in serum, respectively, were 391.34 ± 115.81 (BMI≦25), 520.87 ± 192.41 (25 < BMI≦30), and 745.21 ± 45.24 (BMI≧30). Kruskal-Wallis test showed *χ* ^2^ = 13.59, *P* = 0.001, which means nesfatin-1 in serum has asymptotic significance with BMI variation (Figure 4).

Meanwhile, the nesfatin-1 levels in synovial, respectively, were 88.65 ± 42.7 (BMI≦25), 116.10 ± 55.71 (25 < BMI≦30), and 99.66 ± 46.86 (BMI≧30). Kruskal-Wallis test showed *χ* ^2^ = 2.749, *P* = 0.253, which means no statistical correlation of nesfatin-1 level exists between different BMI.

### 3.5. Nesfatin-1, hsCRP, and PIIANP Levels in Serum of OA Patients and Their Relationship

**Figure 5:**
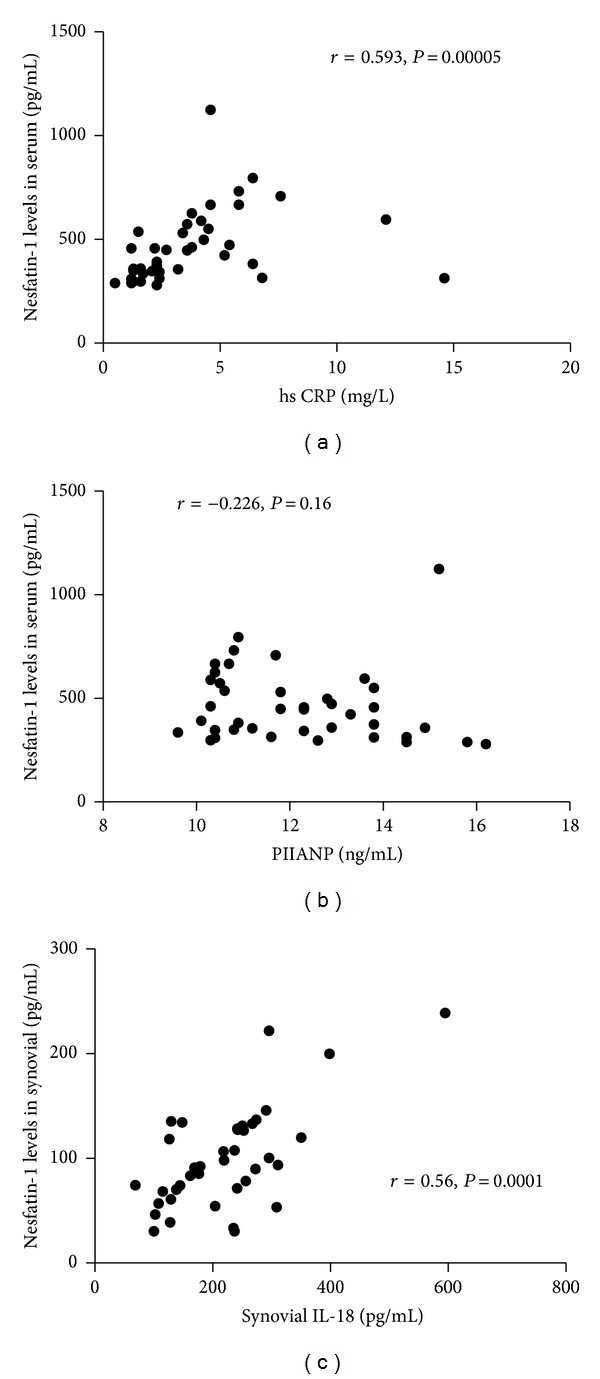
The relationships between serum nesfatin-1 levels and serum hsCRP, PIIANP levels and between synovial nesfatin-1 levels and synovial IL-18 levels. (a) A strong and statistically significant correlation was found between serum nesfatin-1 and hsCRP levels in OA patients (*r* = 0.593, *P* = 0.00005); (b) there was no statistical correlation between serum nesfatin-1 and PIIANP levels (*r* = −0.226, *P* = 0.16); (c) statistically significant correlation was found between synovial nesfatin-1 and IL-18 levels *r* = 0.560, *P* = 0.0017.

A strong and statistically significant correlation was found between serum nesfatin-1 and hsCRP levels in these 40 OA patients [*r* = 0.593, *P* = 0.00005, Table 1 and Figure 5(a)]. The average serum hsCRP level was 3.82 ± 2.88 mg/L. However, there was no statistical correlation between serum nesfatin-1 and PIIANP levels (*r* = −0.226, *P* = 0.16) (Figure 5(b)).

### 3.6. The Relationship between Nesfatin-1 and IL-18 Levels in Synovial from Patients with OA

Statistically significant correlation was found between synovial nesfatin-1 and IL-18 levels [*r* = 0.560, *P* = 0.0017, Table 1 and Figure 5(c)]. The average synovial IL-18 level was 218.99 ± 98.42 pg/mL.

### 3.7. Immunohistochemical Analysis of Nesfatin-1 in OA Knee Cartilage, Osteophytes, and Synovium

**Figure 6:**
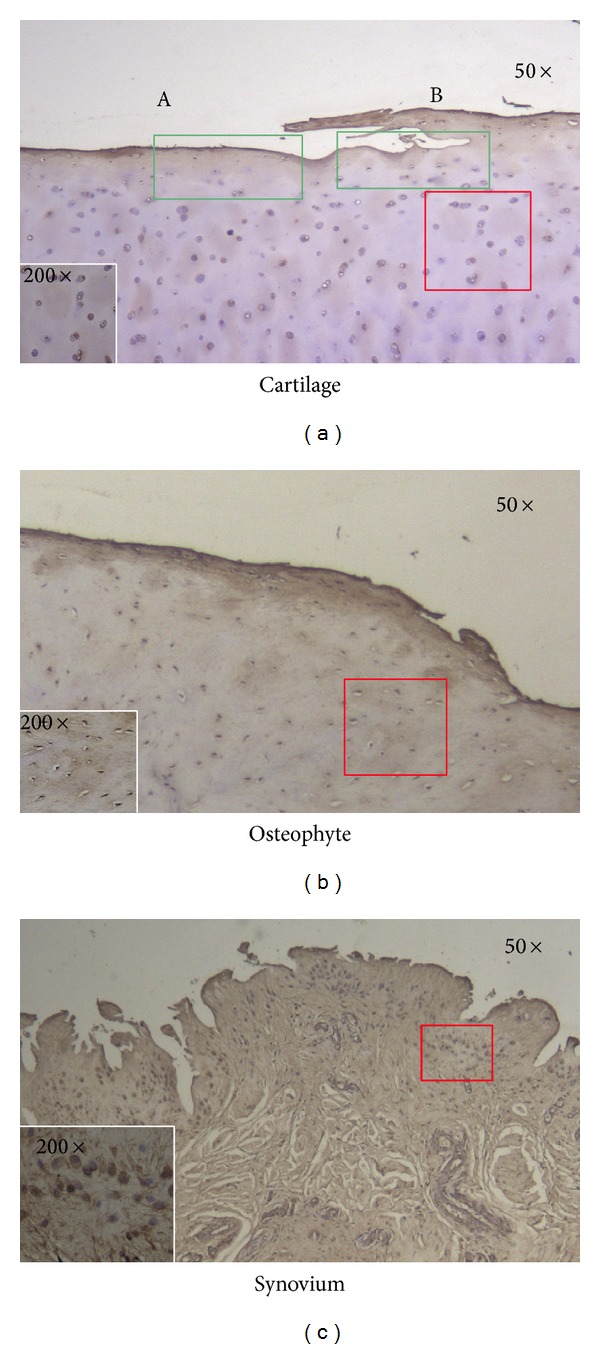
Immunohistochemical analysis of nesfatin-1 in OA knee cartilage, osteophytes, and synovium under 50× and 200× electron microscope fields. All three tissues expressed nesfatin-1, which was especially high in the lesional areas of cartilage (B > A) and the superficial layers of osteophytes and synovium. Chondrocytes, osteophyte cells, and synoviocytes were strongly stained ((a) damaged cartilage area, (b) nondamaged cartilage area).

Staining of representative slides demonstrated that all OA cartilage, osteophyte, and synovium samples contained nesfatin-1. Nesfatin-1 staining was stronger in the lesional cartilage area than in the nonlesional area (B > A). Nesfatin-1 was also present in the synovium and osteophytes, notably in the superficial layer (Figure 6).

## 4. Discussion

It is increasingly apparent that OA is not just a weight-loading disease but is also a multifactorial degenerative joint disease involving metabolic and biochemical factors [26]. Previous studies indicated that adipose tissue, as a metabolic and endocrine organ, secretes many factors, including adipokines leptin, adiponectin, resistin, chemerin, visfatin, and other cytokines [27]. It has been reported that adipokines may be of relevance to pathophysiologic inflammation in OA. For example, adiponectin has been demonstrated to play a protective or deleterious role in OA [28, 29], while serum adiponectin levels were found to be at increased levels in patients with erosive compared with nonerosive OA [30]. Leptin was found to be secreted by osteophytes, synovium, cartilage, and the infrapatellar fat pad [31]. Leptin was the first adipokine to be described (in 1994), and its biological function has been researched relatively thoroughly.

The newly identified anorexigenic protein nesfatin-1 is reported to be ubiquitously expressed in the body [19, 21, 22, 32, 33]. Nevertheless, there are no data regarding the expression and effects of nesfatin-1 in chondrocytes. This study demonstrated (1) nesfatin-1 gene expression in five OA-affected knee articular tissues; (2) increased nesfatin-1 gene expression in patients with hip arthritis compared with individuals with femoral neck fractures; (3) the presence of nesfatin-1 in SF obtained from patients with OA; (4) increased serum levels of nesfatin-1 in patients with OA, as compared to healthy, non-OA individuals; (5) serum nesfatin-1 levels varied with BMI in OA patients; (6) serum nesfatin-1 levels had statistically significant correlation with serum hsCRP, but without PIIANP; and (7) synovial nesfatin-1 levels had statistically significant correlation with IL-18 in synovial fluid.

In the present study, nesfatin-1 was detected in SF from patients with OA, but the levels of nesfatin-1 in serum in patients with OA exceeded those in paired SF samples (*P* < 0.001). This finding demonstrated that circulating levels of nesfatin-1 did not accurately represent the situation in the joint. This is in line with previous observations that serum levels of adipokines such as adiponectin, leptin, and apelin were increased [6, 34]. Nesfatin-1 was secreted by white adipose tissue (WAT), which is widely distributed in the body. However, the joint cavity is a physically isolated space with relatively low WAT levels compared with the body as a whole. Thus, abundant peripheral fat stores may be one of the reasons that nesfatin-1 levels were higher in the bloodstream.

Osteophytes, synovium, cartilage, and the infrapatellar fat pad can secrete adipokines [31, 35]. All five OA-affected knee articular tissues we studied (cartilage, osteophytes, synovium, meniscus, and the infrapatellar fat pad) expressed the nesfatin-1 gene. Yosten and Samson found that nesfatin-1 acts through the central oxytocin system and that its effects can be reversed by pretreatment with an oxytocin receptor antagonist [36]. Because nesfatin-1 was detected in SF from patients with OA and the nesfatin-1 gene was expressed in OA-affected articular tissues, we can speculate that some nesfatin-1 receptor may exist on human chondrocytes, synoviocytes, fat pad adipocytes, or even osteocytes and that nesfatin-1 may penetrate into these tissues, binding to this receptor.

Nesfatin-1 gene expression was higher in OA cartilage, as compared to cartilage from healthy individuals (*P* < 0.05), indicating that nesfatin-1 may be involved in the pathogenesis of OA. Furthermore, serum levels of nesfatin-1 were distinctly higher in in patients with OA than in non-OA individuals (*P* < 0.05). Excessive secretion of adipokines was described as being associated with proinflammatory effects [16, 27, 37] and rheumatoid arthritis [38]. Local inflammation and synovitis have been observed in the pathological course of many patients with OA and animal models of OA. In view of the aforementioned evidence, nesfatin-1 appears to exert a proinflammatory effect in the progress of OA. However, additional studies are required to clarify the contribution of nesfatin-1 to the pathogenesis of OA to determine whether nesfatin-1 has a protective or aggressive role in OA.

Leptin and adiponectin were found to associate with BMI [6]. The similar phenomenon was observed in our research. Synovial nesfatin-1 levels had no linear relation with BMI. However, serum nesfatin-1 levels correlated positively with BMI in OA patients (*χ* ^2^ = 13.59, *P* = 0.001), demonstrating that systemic nesfatin-1 levels may be influenced by the body fat. As inflammatory index, hsCRP has special value in reflecting OA state. We explored the relation between hsCRP and nesfatin-1, which prompts significant positive relation in patients with OA (*r* = 0.593, *P* = 0.00005). A study on peritoneal dialysis patients showed there was a close relationship between proinflammatory cytokines and adipokines, including IL-18 and adiponectin [39]. We concluded that synovial nesfatin-1 and IL-18 levels had positive correlation. Above on, the relationships of nesfatin-1, hsCRP, and IL-18 are of relevance in the inflammatory component of OA. PIIANP is a new molecular marker to predict the progression of articular cartilage damage [40]. PIIANP was found decreasing in OA patients. There was no statistically significant correlation to be found in serum nesfatin-1 and serum PIIANP in our results. However, more deep research is needed in detecting the role in OA progression of these two new molecular markers.

In this study, we detected nesfatin-1 expression in OA knee cartilage, osteophytes, and synovium by IHC staining. In synovium, the number of synoviocytes secreting nesfatin-1 was high, corresponding with the rich WAT in the synovium. This is in accordance with a previous study showing that synovium and the infrapatellar fat pad are major sources of adipokines [31]. Additionally, nesfatin-1 expression was mainly detected in the superficial and border of cartilage and subchondral bone. Nesfatin-1 staining was stronger in the lesional cartilage area than in the nonlesional area. This indicates that nesfatin-1 may play a protective role or respond to the OA pathological process.

Admittedly, this study is limited and further research is required. The samples we investigated were insufficient for conclusive results. Clinical studies with larger sample sizes are urgently needed. It has been reported that the leptin expression levels were related to the grade of cartilage destruction [41]. In our study, we did not compare the relation between nesfatin-1 levels and OA severity. Previous studies of sex differences in patients with OA showed that serum leptin levels were higher in females than in males [31] and that adipokine expression differed between males and females [6].

In conclusion, in the first study of nesfatin-1 in OA, we discovered that human chondrocytes express nesfatin-1, which was also detected in SF from patients with OA. We also confirmed that the serum nesfatin-1 concentration was higher in patients with OA than in healthy, non-OA controls. Nesfatin-1 gene expression was higher in OA cartilage compared with non-OA cartilage. Furthermore, OA cartilage, osteophytes, synovium, meniscus, and infrapatellar fat pads express the nesfatin-1 gene, consistent with the results of IHC of cartilage, osteophytes, and synovium. Taken together, the results of this study highlight a potentially pivotal role of nesfatin-1 in the pathophysiology of OA.

## Figures and Tables

**Table 1:** Characteristics of patients with knee OA and healthy, non-OA controls.

Characteristic	OA (*n* = 40)	Controls (*n* = 25)	p1	p2
Age (years)	66.17 ± 5.82	61.52 ± 6.45	>0.05	
Gender (F/M)	26/14	16/9		
BMI (kg/m^2^)	25.34 ± 2.81	25.06 ± 1.71	>0.05	
S-nesfatin-1 (pg/mL)	466.45 ± 175.83	352.05 ± 137.51	<0.05	
SF-nesfatin-1 (pg/mL)	98.96 ± 47.97	NM		<0.001
hsCRP (mg/L)	3.82 ± 2.88			
PIIANP (ng/mL)	12.17 ± 1.77			
IL-18 (pg/mL)	218.99 ± 98.42			

OA: osteoarthritis; F: female; M: male; BMI: body mass index; S: serum; SF: synovial fluid; NM: not measured.

p1: OA versus controls.

p2: OA SF-nesfatin-1 versus OA S-nesfatin-1.

**Table 2:** Primers for targeted genes.

Gene	GenBank Accession no.	Sequence (5′ to 3′)	Size (bp)	Annealing temp. (°C)
Human nesfatin	NM 005013.2	GCATGGACCACCAAGCTCTTCTAA	117	62
		GTTCCAGATCACTTGTTGCCGCTTT		

Human 18S rRNA	NR 003286	GACTCAACACGGGAAACCTCAC	122	62
		CCAGACAAATCGCTCCACCAAC		
